# Copy number evolution and its relationship with patient outcome—an analysis of 178 matched presentation-relapse tumor pairs from the Myeloma XI trial

**DOI:** 10.1038/s41375-020-01096-y

**Published:** 2020-12-01

**Authors:** James Croft, Sidra Ellis, Amy L. Sherborne, Kim Sharp, Amy Price, Matthew W. Jenner, Mark T. Drayson, Roger G. Owen, Sally Chown, Jindriska Lindsay, Kamaraj Karunanithi, Hannah Hunter, Walter M. Gregory, Faith E. Davies, Gareth J. Morgan, Gordon Cook, Lilit Atanesyan, Suvi Savola, David A. Cairns, Graham Jackson, Richard S. Houlston, Martin F. Kaiser

**Affiliations:** 1 Division of Molecular Pathology, The Institute of Cancer Research, London, UK; 2 Department of Haematology, University Hospital Southampton NHS Foundation Trust, Southampton, UK; 3 Institute of Immunology and Immunotherapy, University of Birmingham, Birmingham, UK; 4 Haematological Malignancy Diagnostic Service, St. James’s University Hospital, Leeds, UK; 5 Gloucestershire Hospitals NHS Foundation Trust, Gloucester, UK; 6 Kent and Canterbury Hospital, Canterbury, UK; 7 University Hospital of North Midlands, Stoke-on-Trent, UK; 8 Derriford Hospital, Plymouth, UK; 9 Clinical Trials Research Unit, Leeds Institute of Clinical Trials Research, Leeds, UK; 10 Perlmutter Cancer Center, NYU Langone Health, New York, NY USA; 11 Leeds Institute of Cancer and Pathology, University of Leeds, Leeds, UK; 12 MRC-Holland, Amsterdam, The Netherlands; 13 Department of Haematology, University of Newcastle, Newcastle Upon Tyne, UK; 14 Division of Genetics and Epidemiology, The Institute of Cancer Research, London, UK; 15 Department of Hematology, The Royal Marsden Hospital, London, UK

**Keywords:** Genetics research, Cancer genomics, Myeloma

## Abstract

Structural chromosomal changes including copy number aberrations (CNAs) are a major feature of multiple myeloma (MM), however their evolution in context of modern biological therapy is not well characterized. To investigate acquisition of CNAs and their prognostic relevance in context of first-line therapy, we profiled tumor diagnosis–relapse pairs from 178 NCRI Myeloma XI (ISRCTN49407852) trial patients using digital multiplex ligation-dependent probe amplification. CNA profiles acquired at relapse differed substantially between MM subtypes: hyperdiploid (HRD) tumors evolved predominantly in branching pattern vs. linear pattern in t(4;14) vs. stable pattern in t(11;14). CNA acquisition also differed between subtypes based on *CCND* expression, with a marked enrichment of acquired del(17p) in *CCND2* over *CCND1* tumors. Acquired CNAs were not influenced by high-dose melphalan or lenalidomide maintenance randomization. A branching evolution pattern was significantly associated with inferior overall survival (OS; hazard ratio (HR) 2.61, *P* = 0.0048). As an individual lesion, acquisition of gain(1q) at relapse was associated with shorter OS, independent of other risk markers or time of relapse (HR = 2.00; *P* = 0.021). There is an increasing need for rational therapy sequencing in MM. Our data supports the value of repeat molecular profiling to characterize disease evolution and inform management of MM relapse.

## Introduction

Multiple myeloma (MM) is caused by the malignant clonal expansion of plasma cells in the bone marrow [1]. Approximately 40% of MM tumors harbor chromosome t(4;14), t(11;14), or t(14;16)/t(14;20) translocations, which result in overexpression of oncogenes (including *WHSC1*/*MMSET*/*NSD2*, *FGFR3*, *CCND1*, *MAF*, and *MAFB*) through juxtaposition to the immunoglobulin heavy-chain locus [2, 3]. Other MM tumors exhibit hyperdiploidy (HRD), which is considered an alternative initiating event. Increased expression of *CCND1* in t(11;14) and HRD and increased *CCND2* expression in t(4;14), t(14;16)/t(14;20), and HRD are unifying downstream consequences of initiating lesions [4–10].

Recent next-generation sequencing projects have provided insight into the sub-clonal complexity of tumor progression in MM [11–14]. However, few recurrent single nucleotide changes have been reported to be associated with disease relapse, notably sub-clonal mutations of cereblon in immunomodulatory drug (IMiD) treated patients. Knowledge about their prognostic impact remains limited [15]. In contrast, several large-scale chromosomal abnormalities, including gain(1q), del(1p), and del(17p) have been shown to be acquired during tumor progression, but their association with tumor subtype, treatment and outcome is not well understood [11, 16]. Recent studies of the relationship between copy number aberrations (CNAs) and progression of MM have largely been based on short-read sequencing or FISH. Importantly, few studies have been performed on patients from a randomized clinical trial and the impact of therapy on acquisition of CNAs remains unclear [15, 16]. To gain insight into the relationship between CNAs and progression of MM we performed virtual karyotyping of all chromosomes and targeted hotspot profiling using digital multiplexed ligation-dependent probe amplification (digitalMLPA) in matched diagnosis–relapse tumor pairs in 178 UK NCRI Myeloma XI trial patients [17]. We show that acquisition of CNAs is strongly correlated with MM subgroups. Additionally, a number of acquired changes have independent predictive value for defining patient outcome.

## Material and methods

### Patients

We studied 178 newly diagnosed MM patients enrolled in UK NCRI Myeloma XI (ISRCTN49407852) for which high quality bone marrow tumor material at both presentation and relapse was available. First outcomes of the trial have been published recently [17]. Briefly, patients were initially randomized to triplet induction with thalidomide (CTD), lenalidomide (CRD), or carfilzomib and lenalidomide (KCRD; transplant-eligible patients only) in combination with cyclophosphamide and dexamethasone. Insufficient responders (partial or minimal response) were randomized to cyclophosphamide, bortezomib and dexamethasone (CVD) vs. no intensification and nonresponders (stable or progressive disease) received CVD. Younger, fitter patients received high-dose melphalan (HDMEL) and autologous stem-cell transplantation, patients were randomized to receive lenalidomide, lenalidomide plus vorinostat, or observation. Maintenance treatment continued until progressive disease in the absence of toxicity (Supplementary Fig. 1).

Median time to progression was 20.7 months (range 3.7–71.9 months) and median follow-up 47.0 months. Baseline characteristics are summarized in Supplementary Table 1. The frequency of chromosomal aberrations detectable in the 178 patients at presentation were representative of the overall trial cohort (Supplementary Table 2) [4].

For all patients CD138-positive tumor cells were immunomagnetically selected at presentation and relapse and quality controlled for tumor cell purity (>95%), DNA and RNA were extracted using QIAGEN (Hilden, Germany) Allprep kits. Additional molecular quality control including longitudinal consistency for IgD deletion status was performed for all cases. Matched identity of presentation-relapse pairs was confirmed using single nucleotide and/or insertion/deletion polymorphisms assayed by digitalMLPA D006-X2 for all samples.

All patients provided written informed consent. The study was approved by the UK National Research Ethics Service, research ethics committees at participating centers and the UK Medicines and Healthcare Products Regulatory Agency.

### Translocation and copy number profiling

Multiplexed qRT-PCR was used to determine t(4;14), t(6;14), t(11;14), t(14;16), and t(14;20) status in tumors, based on the translocation cyclin D (TC) classification system as previously described [5, 18].

Targeted genome wide CNA status at presentation and relapse in each patient was assessed with a newly developed research version of D006-X2 Multiple Myeloma digitalMLPA probemix, as previously described (Supplementary Methods) [19, 20]. Details of each probe and their respective genomic positions are provided in Supplementary Table 3. CNAs involving sex chromosomes were not considered. CNAs were called if ≥50% of probes mapping to an individual gene or genomic region deviated from normal diploid pattern.

### Statistical analyses

Analyses were performed in R version 3.5.2 using “dplyr”, “tidyr”, “stats”, “survival” and “survminer”, “ComplexHeatmap” and “ggpubr” packages. The association between categorical variables was examined using Fishers exact test and between continuous variables using the Wilcoxon signed-rank test. Progression-free survival (PFS) was defined as time from induction randomization to progression, according to International Myeloma Working Group criteria, or death of any cause. Overall survival (OS) was time from induction randomization to death of any cause. Cox proportional hazards (PH) regression was used to estimate univariate and multivariable hazard ratios (HRs) and 95% confidence intervals (CI). To examine the predictive value of evolution in CNA status for subsequent therapy, high-risk CNA were considered as time dependent covariates within the multivariate model. The PH assumption was tested to investigate whether time dependent covariate effects persisted irrespective of time of acquisition (Supplementary Methods). Multivariate covariates included t(4;14), t(14;16), del(1p), gain(1q), del(17p) and treatment pathway (transplant eligible vs. not). This is relevant because, although the median follow-up of this cohort is long, the inherent requirement of a relapse biopsy excludes the longest responders from our analyses as they are still on active trial medication or observation with no evidence of disease progression. Differences between Kaplan–Meier survival curves was assessed using the log-rank test. A two-sided *P* value of ≤0.05 was considered statistically significant.

## Results

### Acquisition of new CNAs is a feature of relapse

Relapse was associated with the acquisition of new CNAs in 87.1% of tumors, most (73.2%) being large-scale chromosome changes. Across all patients, relapse was associated with a higher number of CNAs: median 11.5 (range 0–34) vs. median 12 (range 0–29) (*P* = 0.0058). As previously well-documented [16, 21], IGH translocations were clonal at presentation and their status did not change at relapse.

**Fig. 1:**
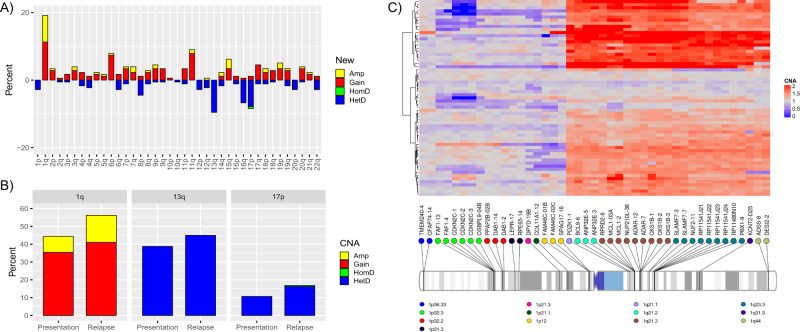
Recurrent chromosomal and sub-chromosomal CNA changes at relapse. **A** Frequency of CNAs emerging at relapse affecting chromosome arms, including new gain (red), amplification (yellow), heterozygous deletion (blue), or homozygous deletion (green) plotted as a bar graph. **B** Bar graph showing frequency of 1q, 13q, and 17p CNAs, including gain (red), amplification (yellow), heterozygous deletion (blue), or homozygous deletion (green), at presentation and relapse. **C** Heat map of evolving chromosome 1 CNAs at relapse, 63 tumors with change in this region at relapse. On the left (*Y*-axis) dendrogram representing unsupervised clustering analysis of emerging CNAs in areas interrogated by digitalMLPA probes, which are annotated with gene names and chromosomal location below (*X*-axis). Change per probe and tumor color-coded as per scale representing normalized digitalMLPA copy number ratios with 1.0 equivalent to normal/diploid.

The most frequent chromosomal changes associated with relapse were gain or amplification of 1q (19%), del(13q) (10%), gain or amplification of 11q (9%) and del(17p)/*TP53* in (8%) (Fig. 1; Supplementary Table 2). Although not common, some clonal CNAs detectable at presentation were not detectable at relapse, in particular gain of odd numbered chromosomes in HRD tumors (Supplementary Fig. 2).

### Evolution of sub-chromosomal aberrations

In contrast to 1q gain, which generally involved the whole chromosomal arm, clonal 1p deletions tended to be sub-chromosomal (Fig. 1): 1p32.3 deletion (9/178; 5.1%) implicating *CDKN2C* or 1p12 implicating *FAM46C* (4/178; 2.2%). In nine of 22 tumors with 1p12 (*FAM46C*) deletion at diagnosis, the deletion was not clonally detectable at relapse; in contrast all but one of the 8 presentation 1p32.3 (*CDKN2C*) deletions were detectable at relapse.

Focal gain at relapse also affected 8q24.21, involving the *MYC* locus, in 10/178 (5.6%) tumors. In three of 15 tumors with *MYC* gain at presentation, reversion to diploid status was a feature of relapse.

Sub-chromosomal CNAs involving IMiD response genes—*CRBN* (3p26.2), *IKZF1* (7p12.2), *IKZF3* (17q12), and *IRF4* (6p25.3)—were infrequent (Supplementary Fig. 3). Most were detectable in tumors relapsing off therapy (no maintenance); only 2 out of 10 were a feature of tumors from patients in receipt of lenalidomide maintenance (1 deletion of *CRBN*; 1 gain *IRF4*).

### Evolution of driver copy number aberrations

We next examined for clonal emergence at relapse of sub-clonal changes at diagnosis, focusing on the most frequent drivers—gain(1q) and del(17p). We and others showed before that calling of CNAs using conservative high confidence MLPA cutoff values detects clonal infiltration equivalent to about >20% by FISH, but that calling of minor sub clones is also feasible [4]. Thirty percent of patients’ tumors with clonally detectable 1q gain at relapse had a detectable, potential minor sub-clonal gain(1q) at diagnosis and 50% of del(17p) relapse tumors had potential sub-clonal del(17p) at diagnosis (Supplementary Fig. 4). Progressive clonal expansion of 1q positive tumors was a feature of 17.5% gain(1q) cases, whereby gain at diagnosis evolved into tetraploidy of 1q (amp(1q)) at relapse. Two of 15 tumors with gain of *MYC* at diagnosis progressed to amplification at relapse. This was also a feature with gain of 15q (4.4%) and 19p (2.3%), albeit at low frequency.

**Fig. 2:**
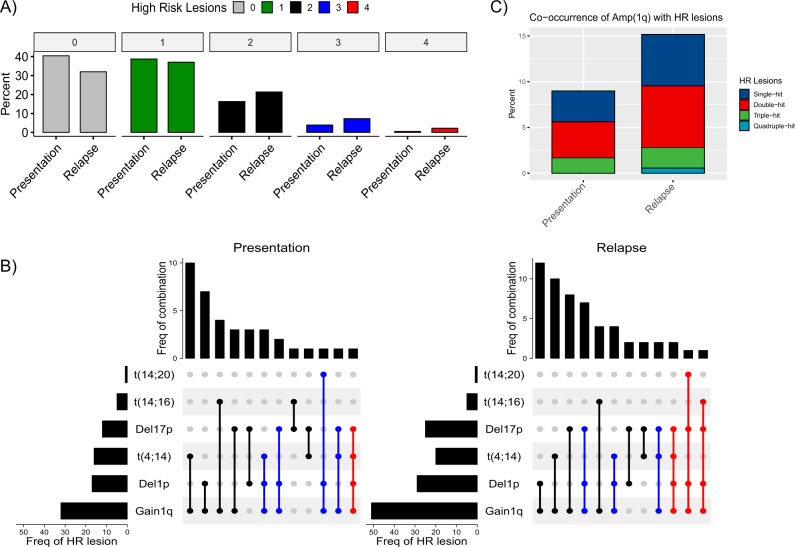
Positive selection of co-occurrence of high-risk lesions. **A** Frequency bar chart of tumors characterized by number of high-risk markers, ranging from no (0) high-risk marker to co-occurrence of 4 markers. **B** Upset plots of Presentation (left) and Relapse (right) tumors, each showing frequency of individual high-risk lesions (left), type of combination of lesions (center lines with dots indicating presence of individual lesion) and frequency of specific combination of markers (top). **C** Bar chart showing the overall frequency of amp(1q) between presentation and relapse (overall bar height) and proportion of amp(1q) tumors showing 1q as the only high-risk aberration (single hit), or in combination with one or more other high-risk markers (double hit to quadruple hit).

We and others have demonstrated that patients with double or triple hit tumors (i.e., 2+ high-risk aberrations t(4;14), t(14;16), t(14;20), gain(1q), or del(17p)) have an especially poor prognosis [4]. It was noteworthy that some tumors with 0 or 1 lesions progressed to carrying double, triple and quadruple hits at relapse (Fig. 2). Amplification 1q has recently been proposed as an additional independent marker of high risk by some researchers [22]. In our study around two thirds of amp(1q) tumors were “double hit”, i.e., also carried t(4;14), t(14;16), t(14;20), or del(17p). Of all 178 relapsed tumors, nearly 10% carried both amp(1q) and “double hit” genetic features (Fig. 2).

### Impact of subtype on copy number profile

Given the biological heterogeneity of MM we examined the relationship between CNAs and disease progression by subtype: those with IG translocations (t(4;14), t(11;14), t(14;16)/t(14;20)), and those with HRD, sub-grouped into those with and without gain of 11, in analogy to Translocation and Cyclin D (TC) classification (Supplementary Table 2) [4, 5].

**Fig. 3:**
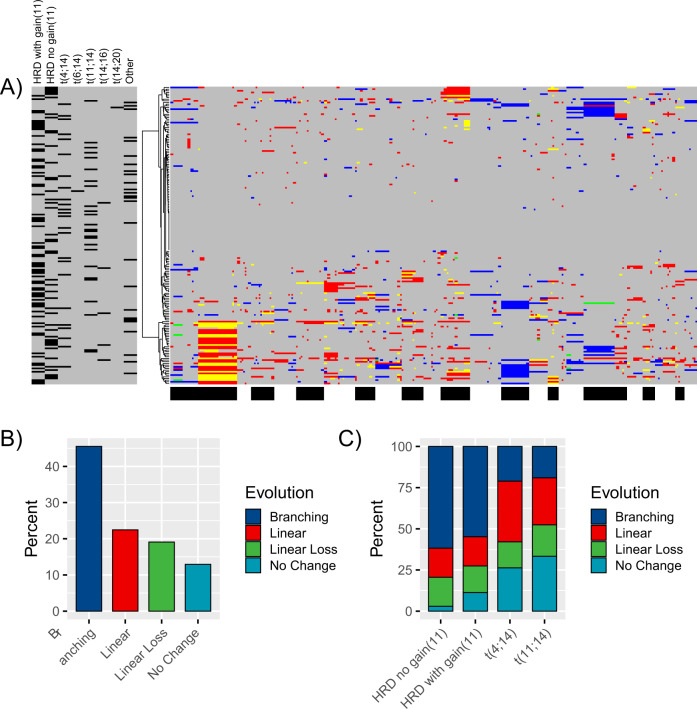
Relationship between CNAs at presentation and relapse and MM subtype. **A** Co-occurrence in evolution of novel CNAs at relapse in context of pathogenetic lesions (legend left side; dark bar = present, gray = absent; legend bottom: black and white bands representing chromosomal mapping of digitalMLPA probes chr1-22 from left to right in ascending order of genomic position) displayed in a heat map with unsupervised clustering for evolving CNAs. **B** Frequency of evolutionary patterns across all tumors. **C** Frequency of evolutionary patterns per major molecular MM subgroups.

**Fig. 4:**
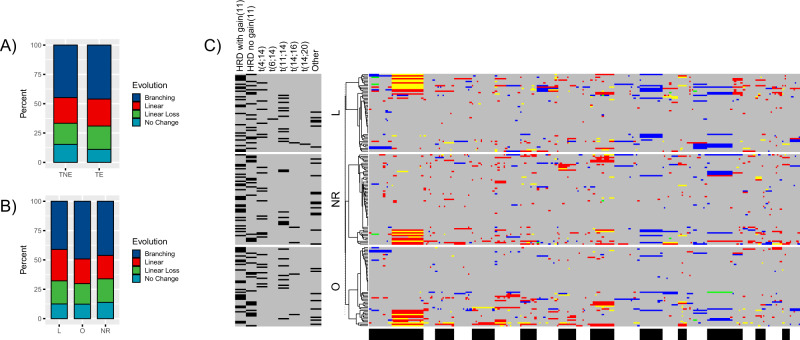
Evolutionary patterns in context of trial treatment. **A** Comparison of frequencies of evolutionary patterns between transplant eligible (TE; younger, fitter patients) and transplant non eligible (TNE). **B** Comparison of frequencies of evolutionary patterns for patients randomized to lenalidomide (L) maintenance, observation (O) or those not randomized (NR) (**C**) Heatmaps of CNA changes at relapse for tumors from patients randomized to lenalidomide (L) maintenance, observation (O) or those not randomized (NR). For each group separately unsupervised clustering on CNA changes was performed and a dendrogram is shown on the left of each heat map. Legend on left side provides context on molecular background lesions (dark bar = present, gray = absent). Legend at bottom: black and white bands representing chromosomal mapping of digitalMLPA probes chr1-22 from left to right in ascending order of genomic position.

Emerging CNA patterns at relapse were classified as branching (45.5% of all tumors), linear (22.5%), linear loss (19.1%), and stable (12.9%) (Fig. 3). In HRD the majority of tumors showed evidence of branching evolution (57.3%) (Fig. 3). In contrast, linear evolution dominated (36.8%) t(4;14) MM. t(11;14) tumors primarily showed either no CNA change at relapse (stable; 33.3%), or linear evolution (28.6%) (Fig. 3). Importantly, there was no relationship between acquisition of CNAs and either HDMEL or lenalidomide maintenance therapy (Fig. 4).

Both del(13) (94.7%) and gain/amp (1q) (68.4%) were seen at high frequency in t(4;14) MM at presentation, and the CNAs were seen at even higher frequencies in relapse tumors at 100% and 79.0%, respectively (Supplementary Fig. 5). Moreover, at relapse del(17p) was a feature of 31.6% of t(4;14) tumors (Supplementary Fig. 5). In contrast, acquisition of 1q, 2p, 8q, 9p, 9q, or 6q CNAs, the changes occurring in t(11;14), were not common; each of these aberration was acquired in <5% of t(11;14) relapsed tumors (Fig. 3). Due to their low frequency, as expected, t(14;16) (two patients) and t(14;20) (one patient) tumors are not discussed; sequential CNA analysis providing limited information regarding subgroup evolutionary trajectory. Baseline and relapse CNAs for t(14;16) and t(14;20) shown in Supplementary Fig. 6.

Finally, we examined the relationship between CNA change and MM subtypes defined by *CCND* expression; *CCND1* (D1), *CCND2* (D2), or both (D1 + D2) (Supplementary Fig. 7). Acquisition of del(17p) was associated primarily with D2 subtype (Supplementary Fig. 8). Specifically, acquired del(17p) at relapse was a feature of 14.6% of D2, 12.0% of D1 + 2, and 5.1% of D1 tumors. Overall, acquisition of new lesions was less frequent in HRD with gain(11), characterized by D1 expression, vs. other HRD tumors (Supplementary Fig. 9).

### Associations with patient outcome

Gain(1q) (HR = 2.23; *P* < 0.001), del(1p)/*CDKN2C* (HR = 1.81; *P* = 0.046), gain(8q)/*MYC* (HR = 1.87; *P* = 0.033), and del(17p) (HR = 2.95; *P* < 0.001) considered as time dependent variables, i.e., including their acquisition at relapse, were all independently associated with shorter OS (Table 1; Supplementary Fig. 10; Supplementary Table 4).

**Table 1:** Multivariate Cox regression analysis of risk factors for OS, where CNAs represent time dependent covariates.

	HR	95% CI	*P* value
t(4;14)	1.25	(0.62–2.54)	0.53
t(14;16)	1.75	(0.66–4.60)	0.26
Gain(1q)	2.23	(1.39–3.57)	0.0008
Del(1p)/*CDKN2C*	1.81	(1.01–2.26)	0.046
Gain(8q)/*MYC*	1.87	(1.05–3.31)	0.033
Del(17p)	2.95	(1.67–5.20)	0.0001
TNE	2.30	(1.41–3.75)	0.0008

*HR* hazard ratio, *95% CI* 95% confidence interval.

**Fig. 5:**
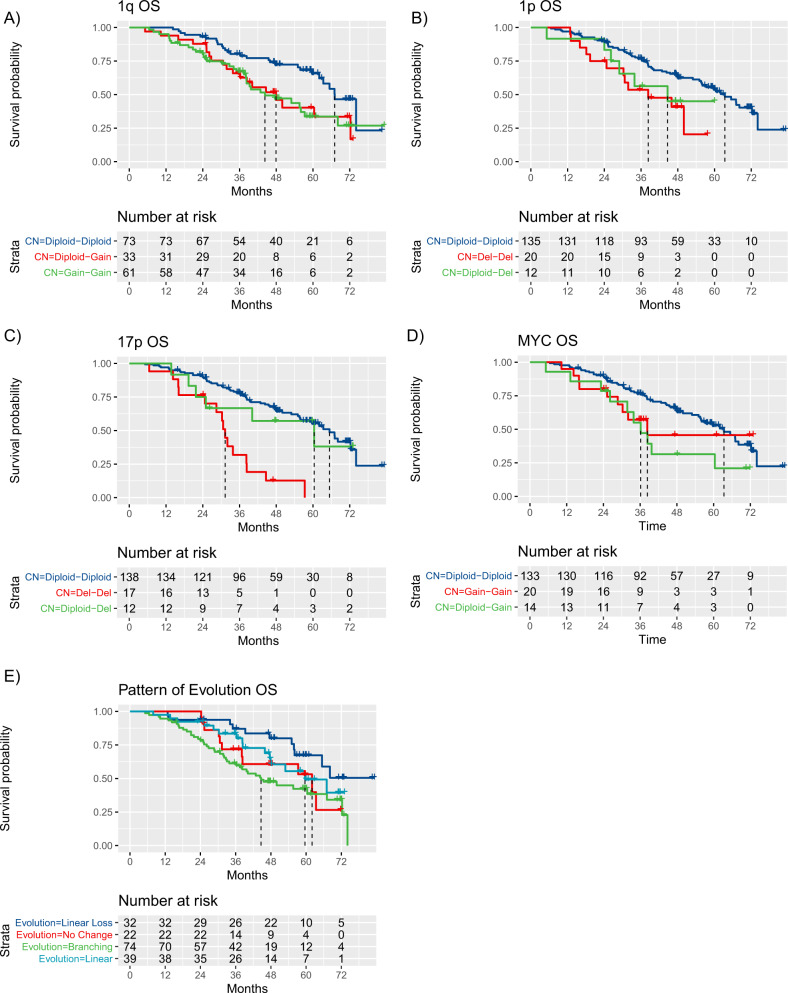
Relationship between emerging CNAs, evolutionary trajectories, and patient outcome. **A** Kaplan–Meier plot for overall survival in context of presence of CNAs for 1q. Equivalent plots for aberrations of (**B**) deletion of 1p21 (*CDKN2C*) (**C**) gain of 8q21 (*MYC*) (**D**) deletion of 17p (*TP53*) (**E**) evolutionary pattern. As per individual color-coded label for **A**–**D**, curves represent patients with tumors remaining diploid at both time points, changing from diploid to abnormal or showing abnormality at both time points.

CNAs were stratified by time point of acquisition, i.e., “Gain-Gain” for those with stable gain and “Diploid-Gain” for those with evolution of gain(1q) at relapse. Gain(1q) from presentation and evolution of new gain(1q) at relapse were both associated with significantly shorter OS compared to normal 1q copy number (HR 2.11; *P* = 0.0040 and HR 2.00; *P* = 0.021, respectively). Median OS was 44.3 vs. 47.9 vs. 67.1 months for gain(1q) at diagnosis, new gain(1q) at relapse and normal 1q copy number at both time points, respectively (log-rank *P* = 0.007) (Fig. 5).

Acquired del(17p), del(1p/*CDKN2C*) or gain(8q/*MYC*) at relapse were not clearly associated with a significantly worse OS in subgroup analyses, although our power to demonstrate a relationship was naturally limited by low frequency of these CNAs. Median OS was 31.3 vs. 60.5 vs. 65.4 months for del(17p) at diagnosis, emergence at relapse and absence at both, respectively (log-rank *P* < 0.001) (Fig. 5). Median OS was 38.3 vs. 44.6 vs. 63.4 months for del(1p/*CDKN2C*) at diagnosis, emergence at relapse or absence, respectively (log-rank *P* = 0.03) (Fig. 5); and for gain(8q/*MYC*), 38.3 vs. 36.2 vs. 63.4 months, respectively (log-rank *P* = 0.07) (Fig. 5).

Of interest, type of evolutionary pattern was associated with OS; branching evolution was significantly associated with the shortest and linear loss with the longest OS (HR 2.61, *P* = 0.0048), with median OS being 44.6 vs. 59.6 vs. 62.0 vs. 68.1 months for branching, linear, no change and linear loss respectively (log-rank *P* = 0.02) (Fig. 5).

## Discussion

Our analysis demonstrates that progression of MM is characterized by emergence of clones with additional large-scale chromosomal changes, commonly involving 1q. Gain or amp(1q) frequently co-evolves with other CNAs, either implicating 1q directly in their genesis as per “jumping 1q” hypothesis, or highlighting 1q as a region providing clonal advantage for genetically instable tumors [23]. While a number of genes on 1q have been proposed as drivers, our study does not provide data to make a specific inference [8, 24–27]. Our findings also suggest progression of MM is associated with positive selection of sub-chromosomal loss of 1p, implicating *CDKN2C*, and thereby indirectly CDK4/6 in conferring clonal survival benefit at progression. We also identified recurrent sub-chromosomal gain at relapse of an area to which *MYC* maps (chr8q24.21), in line with its driver role in B-cell malignancies [28, 29].

By considering MM molecular subtype, we highlight differences in evolution trajectories, particularly between HRD and IG translocated MM, but also between t(4;14) and t(11;14) and/or between *CCND2* and *CCND1* driven tumors [5]. Of note, there is a marked enrichment of acquisition of del(17p) at relapse in *CCND2* driven tumors which has, to our knowledge, not been described before. These differences are features of subtypes irrespective of therapy. Our findings are in keeping with published observations for evolution of pre-therapeutic mutational patterns but extend these in context of therapy and relapse [30–32]. We also describe, to our knowledge for the first time, an association between CNA evolution pattern and subsequent outcome. Whether individual CNA evolution pattern has independent clinical relevance and could be included in management considerations at relapse requires further investigation.

We identified only a low frequency of CNAs to which IMiD response genes map. In conjuncture with published somatic SNV data, results suggest mutation of these genes per se is not the major determinant of acquired resistance to lenalidomide, or other members of this class of agent in MM [12, 14–16, 33–35].

We demonstrate, to our knowledge for the first time, in a randomized controlled trial that acquisition of gain(1q) at relapse is independently and strongly associated with inferior OS [36]. Although findings are also indicative for del(17p), del(1p/*CDKN2C*) and gain(8q/*MYC*), our power to robustly assert clinical relevance of acquired lesions at relapse is inevitably limited by the lower frequency of these CNAs [37]. Clinical evaluation of these markers at relapse is technically feasible in most developed healthcare systems, but currently not widely recommended [38–40].

It is unknown whether sub-clones residing outside of the standard iliac crest bone marrow sampling area at diagnosis are the main source of clonal evolution detected at relapse or whether these predominantly emerge through ongoing genomic instability [12]. Multi-region bone sampling in MM is associated with significant risk and morbidity, making its implementation as part of standard care very challenging. Functional bone marrow soft tissue imaging techniques such as diffusion-weighted whole body MRI and/or molecular techniques including circulating tumor DNA profiling are promising methods in development which may contribute to diagnostic assessment of spatial MM heterogeneity in the future. However, sensitivity, precision and clinical relevance of minor sub-clone detection from a single time point for these methods remains to be established, in particular if results differ from those of parallel bone marrow sampling, before informing clinical management [41–44].

All patients received uniform trial treatment, in particular providing insight into thalidomide and lenalidomide associated CNA changes. However, patients also received low-doses of the oral alkylator cyclophosphamide during induction, which may have specific impact on CNA changes, potentially limiting generalizability of our findings.

In addition, molecular information generated with a targeted tool like digitalMLPA is inherently focused and does not discriminate complex processes potentially underlying identified CNAs, such as chromotripsis or chromoplexy, which are detectable by discovery tools such as whole genome sequencing, or single nucleotide variants captured by myeloma specific CNA/sequencing panels [29, 45–47]. Our data also cannot reflect accompanying changes of the tumor microenvironment, which have been implicated in myeloma progression [48]. However, digitalMLPA is in development for clinical diagnostic application, offers sensitive CNA information specific to myeloma from low tumor DNA quantities at high throughput. It requires limited computational infrastructure and uses standardized analysis algorithms, thus overcoming the significant limitations of conventional FISH analysis [20]. As our study demonstrates, digitalMLPA is suitable for longitudinal intra-individual tracking and provides a standardized and accessible method for cross-study validation and putative implementation in molecularly stratified prospective clinical trials.

Genetic re-profiling at relapse in MM is currently not widely recommended and, as a consequence, often not reimbursed [10, 39, 49]. Whilst treatment options for relapsed disease were until recently very limited, a range of therapeutic regimens with varying intensity are now approved and available, requiring better tools for clinical decision making at relapse [50–53]. Adapting first-line therapy and its intensity to individual tumor risk markers in MM is now a key focus of research, with multiple genetically stratified prospective clinical trials currently ongoing [10, 54, 55]. The debate about optimal tailoring of second line treatment is likely to intensify with increasing therapeutic options. Our study demonstrates not only the frequency of emerging high-risk CNAs at MM relapse, but also the unmet clinical need of patients with chromosomally evolving MM. These patients should ideally be recognized early during relapse and prospective clinical trials investigating longitudinal management strategies adapted to dynamic risk profiling, designed [56].

Our findings strongly support repeated tumor molecular analysis in MM in context of modern treatment, even in circumstances where only selected markers such as 1q can be tested, as a means of tailoring patient treatment beyond first-line therapy.

## Supplementary information

Supplementary Methods

Supplementary Tables

Supplementary Figures
