# Supplementary material for: Copy number evolution and its relationship with patient outcome—an analysis of 178 matched presentation-relapse tumor pairs from the Myeloma XI trial
Source: Leukemia. 2020 Dec 1;35(7):2043–53. doi: 10.1038/s41375-020-01096-y (PMC8257500; doi:10.1038/s41375-020-01096-y)
Supplement: Supplementary file 1 — Supplementary Methods [file 41375_2020_1096_MOESM1_ESM.docx]

**SUPPLEMENTARY METHODS**

**DigitalMLPA panel**

An earlier version of the research digitalMLPA panel used here (D006-X2) has been described previously (<https://doi.org/10.1016/j.jmoldx.2018.06.004>). Briefly, the panel contains 282 target copy number probes which interrogate regions recurrently affected by CNAs in MM as well as one probe specific for *BRAF* V600E mutation. Furthermore, 96 reference probes hybridizing to relatively MM copy number stable regions, 45 input DNA and assay quality control, six X and Y chromosome-specific and 39 pairs of SNP probes for sample identification and detection of sample contamination. Reference probes were used for data normalization and, together with a subset of the target probes, for identification of large chromosome gains and losses. A subset of probes is also defined as a karyotyping set and covers 194 different loci, capturing telomeric, centromeric, and mid-chromosome arm regions for all chromosomes.

**CNA calling**

The following discrete cut-offs were used to call CNA from normalised digitalMLPA probe values; amplification >1.7; gain >1.2 ≤1.7; diploid >0.75 ≤1.2; heterozygous deletion >0.25 ≤0.75 and homozygous deletion ≤0.25. A majority rule (CNA present in ≥50% of relevant probes) was used to determine CNA per gene, chromosome band and chromosome arm.

Amplification was called before gain, homozygous before heterozygous deletion. For example the *CKS1B* gene has 3 probes (covering exons 1, 2 and 3). To call amplification of *CKS1B*, ≥ 2 probes were required to have normalised values of >1.7. To call gain of *CKS1B*, ≥ 2 probes were required to have normalised values >1.2 and not meet the criteria of amplification.

Whole chromosome CNA requires the long and short arms to each show said CNA as per the majority rule. Hyperdiploidy was defined as gain of 2 or more of chromosomes 3,5,7,9,11,15,19 and 21.

**Multivariate regression analysis of OS using time dependent covariates**

Calculated using the “survival” package in R version 3.5.2. The occurrence of high risk CNA per tumor were recorded at diagnosis and relapse. The ‘tmerge’ function was used to record CNA status over two time intervals; months from induction randomisation to relapse (PFS) and months from relapse to death of any cause (difference between OS and PFS). ‘tstart’ and ‘tstop’ denoting time in months at the beginning and end of each time interval respectively. High risk IgH translocations and transplant eligibility status were stable over both time intervals. The proportional hazards (PH) assumption was tested using ‘cox.zph’ requiring a non-significant relationship between Schoenfeld’s residuals and time to be considered true. Cox.zph results were plotted to demonstrate HR over time.
