# Supplementary material for: Copy number evolution and its relationship with patient outcome—an analysis of 178 matched presentation-relapse tumor pairs from the Myeloma XI trial
Source: Leukemia. 2020 Dec 1;35(7):2043–53. doi: 10.1038/s41375-020-01096-y (PMC8257500; doi:10.1038/s41375-020-01096-y)
Supplement: Supplementary file 2 — Supplementary Tables [file 41375_2020_1096_MOESM2_ESM.docx]

**SUPPLEMENTARY TABLES**

**Supplementary Table 1.** Baseline characteristics of 178 Myeloma XI patients

|  | | | 178 MXI patients | |
| --- | --- | --- | --- | --- |
| Sex, n (%) | F |  | **47** | (26.4) |
|  | M |  | **131** | (73.6) |
| Age, mean (SD) | mean |  | **64.9** | (9.7) |
| n (%) | <=75 |  | **160** | (89.9) |
|  | 76-80 |  | **15** | (8.4) |
|  | >80 |  | **3** | (1.7) |
| WHO, n (%) | 0 |  | **58** | (32.6) |
|  | 1 |  | **72** | (40.4) |
|  | 2 |  | **27** | (15.2) |
|  | 3 |  | **12** | (6.7) |
|  | NA |  | **9** | (5.1) |
| PP, n (%) | IgA |  | **54** | (30.3) |
|  | IgD |  | **2** | (1.1) |
|  | IgG |  | **98** | (55.1) |
|  | IgM |  | **1** | (0.6) |
|  | LCO |  | **22** | (12.4) |
| LC, n (%) | Kappa |  | **118** | (66.3) |
|  | Lambda |  | **59** | (33.1) |
| Hb, mean (SD) |  |  | **105.9** | (19.4) |
| plt, mean (SD) |  |  | **226.3** | (93.5) |
| neut, mean (SD) |  |  | **3.8** | (2.3) |
| Creat, mean (SD) |  |  | **98.7** | (52.3) |
| Ca, mean (SD) |  |  | **2.5** | (0.3) |
| Albumin, mean (SD) |  |  | **34.7** | (6.6) |
| LDH, mean (SD) |  |  | **326.7** | (287.3) |
| B2M, mean (SD) |  |  | **5.3** | (6.6) |
| ISS, n (%) | I |  | **40** | (22.5) |
|  | II |  | **85** | (47.8) |
|  | III |  | **46** | (25.8) |
|  | NA |  | **7** | (3.9) |
| Transplant Eligible, n (%) | | | **100** | (56.2) |
| Induction, n (%) | CRD |  | **34** | (34.0) |
|  | CTD |  | **55** | (55.0) |
|  | KCRD |  | **11** | (11.0) |
| Maintenance, n (%) |  | Len | **21** | (21.0) |
|  |  | Len + Vorinostat | **11** | (11.0) |
|  |  | Observation | **31** | (31.0) |
|  |  | NR | **37** | (37.0) |
| Transplant Non Eligible, n (%) | | | **78** | (43.8) |
| Induction, n (%) | CRDa |  | **43** | (55.1) |
|  | CTDa |  | **35** | (44.9) |
| Maintenance, n (%) |  | Len | **21** | (26.9) |
|  |  | Len + Vorinostat | **3** | (3.8) |
|  |  | Observation | **26** | (33.3) |
|  |  | NR | **28** | (35.9) |

**Supplementary Table 2:** New chromosomal CNA evolution at relapse >5% frequency overall, and split up per molecular sub-group

|  |  | New Chromosome Arm CNA >5% | | | | | | | | | | | | | | | | New Focal CNA >5% | | | |
| --- | --- | --- | --- | --- | --- | --- | --- | --- | --- | --- | --- | --- | --- | --- | --- | --- | --- | --- | --- | --- | --- |
|  |  | **Gain/Amp**  **(1q)** | | **Del**  **(13)** | | **Gain/Amp**  **(11q)** | | **Del**  **(17p)** | | **Gain/Amp**  **(6p)** | | **Del**  **(16q)** | | **Gain/Amp**  **(15q)** | | **Gain/Amp**  **(19p)** | | **Gain/Amp**  **(*MYC*)** | | **Del**  **(*CDKN2C*)** | |
|  |  | **n** | % | **n** | % | **n** | % | **n** | % | **n** | % | **n** | % | **n** | % | **n** | % | **n** | % | **n** | % |
| All patients | **n** (%) | | | | | | | | | | | | | | | | | | | | |
| Total | **178** (100) | **34** | 19.1 | **17** | 9.6 | **16** | 9.0 | **15** | 8.4 | **14** | 7.9 | **12** | 6.7 | **11** | 6.2 | **9** | 5.1 | **10** | 5.6 | **9** | 5.1 |
| Molecular sub-groups (information available for all 178 patients) | | | | | | | | | | | | | | | | | | | | | |
| t(4;14) | **19** (10.7) | **2** | 10.5 | **1** | 5.3 | **0** | 0.0 | **3** | 15.8 | **0** | 0.0 | **1** | 5.3 | **0** | 0.0 | **1** | 5.3 | **0** | 0.0 | **0** | 0.0 |
| t(11;14) | **21** (11.8) | **0** | 0.0 | **1** | 4.8 | **0** | 0.0 | **1** | 4.8 | **0** | 0.0 | **1** | 4.8 | **0** | 0.0 | **0** | 0.0 | **1** | 4.8 | **1** | 4.8 |
| t(14;16) | **2** (1.1) | **1** | 50.0 | **1** | 50.0 | **0** | 0.0 | **0** | 0.0 | **1** | 50.0 | **0** | 0.0 | **0** | 0.0 | **1** | 50.0 | **0** | 0.0 | **0** | 0.0 |
| t(14;20) | **1** (0.6) | **0** | 0.0 | **0** | 0.0 | **0** | 0.0 | **1** | 100.0 | **0** | 0.0 | **1** | 100.0 | **0** | 0.0 | **0** | 0.0 | **1** | 100.0 | **0** | 0.0 |
| HRD with gain(11) | **62** (34.8) | **12** | 19.4 | **7** | 11.3 | **2** | 3.2 | **5** | 8.1 | **6** | 9.7 | **3** | 4.8 | **5** | 8.1 | **2** | 3.2 | **4** | 6.5 | **4** | 6.5 |
| HRD without gain(11) | **34** (19.1) | **11** | 32.4 | **3** | 8.8 | **11** | 32.4 | **2** | 5.9 | **3** | 8.8 | **4** | 11.8 | **5** | 14.7 | **5** | 14.7 | **2** | 5.9 | **2** | 5.9 |
| IgH transloc and HRD | **13** (7.3) | **3** | 23.1 | **2** | 15.4 | **0** | 0.0 | **0** | 0.0 | **0** | 0.0 | **0** | 0.0 | **0** | 0.0 | **0** | 0.0 | **0** | 0.0 | **2** | 15.4 |
| Other | **26** (14.6) | **5** | 19.2 | **2** | 7.7 | **3** | 11.5 | **3** | 11.5 | **4** | 15.4 | **2** | 7.7 | **1** | 3.8 | **0** | 0.0 | **2** | 7.7 | **0** | 0.0 |
| CCND groups (information available for 158/178 patients) | | | | | | | | | | | | | | | | | | | | | |
| D1 | **78** (49.4) | **13** | 16.7 | **8** | 10.3 | **3** | 3.8 | **4** | 5.1 | **3** | 3.8 | **3** | 3.8 | **1** | 1.3 | **1** | 1.3 | **6** | 7.7 | **4** | 5.1 |
| D1 + 2 | **25** (15.8) | **5** | 20.0 | **2** | 8.0 | **6** | 24.0 | **3** | 12.0 | **5** | 20.0 | **3** | 12.0 | **3** | 12.0 | **1** | 4.0 | **1** | 4.0 | **1** | 4.0 |
| D2 | **55** (34.8) | **8** | 14.5 | **5** | 9.1 | **3** | 5.5 | **8** | 14.5 | **3** | 5.5 | **5** | 9.1 | **3** | 5.5 | **5** | 9.1 | **2** | 3.6 | **1** | 1.8 |

**Supplementary Table 3.** **digitalMLPA D006-X2 probes.** Probe type: T=Tumor for probes mapping to areas/genes specifically frequently affected by CNAs in myeloma, K=Karyotype for probes that are distributed at relatively similar distance across the genome, to produce a ‘virtual karyogram’, R=Reference for probes mapping to areas less frequently affected by CNAs in myeloma and used for normalization processes. Numbers after gene names refer to exons of respective genes.

| hg19 location | gene  (probe number) | type | hg19 location | gene  (probe number) | type | hg19 location | gene  (probe number) | type | hg19 location | gene  (probe number) | type | hg19 location | gene  (probe number) | type |
| --- | --- | --- | --- | --- | --- | --- | --- | --- | --- | --- | --- | --- | --- | --- |
| 1p36.33 | TMEM240 (S013173) | K | 2p11.2 | REEP1 (S011028) | K/R | 5q31.3 | PCDHAC1 (S011408) | T | 8p23.3 | FBXO25 (S013012) | K | 11p15.5 | DEAF1 (S013410) | K |
| 1p36.33 | CFAP74 (S013039) | K | 2q11.1 | PROM2 (S011030) | K/R | 5q31.3 | PCDHAC2 (S011474) | T | 8p23.3 | CLN8 (S013029) | K | 11p14.3 | ANO5 (S011081) | K |
| 1p32.3 | FAF1 (S011387) | T | 2q24.3 | SCN1A (S011031) | K/R | 5q31.3 | PCDHB2 (S011475) | T | 8p23.1 | GATA4 (S011489) | T | 11p14.3 | ANO5 (S011497) | K |
| 1p32.3 | FAF1 (S011458) | T | 2q32.2 | COL3A1 (S011467) | K/R | 5q31.3 | PCDHB10 (S011409) | T | 8p21.3 | GFRA2 (S011065) | K | 11q12.3 | BEST1 (S011082) | K |
| 1p32.3 | CDKN2C (S011456) | T | 2q37.3 | CAPN10 (S013042) | K/R | 5q31.3 | SLC25A2 (S011476) | T | 8p21.3 | TNFRSF10B (S011418) | T | 11q13.3 | CCND1 (S011498) | T |
| 1p32.3 | CDKN2C (S013428) | T | 2q37.3 | KIF1A (S012999) | K/R | 5q31.3 | PCDHGA11 (S011407) | T | 8p21.3 | TNFRSF10A (S011417) | T | 11q13.3 | CCND1 (S011423) | T |
| 1p32.3 | CDKN2C (S011457) | T | 3p26.2 | TRNT1 (S012953) | K | 5q32 | SH3TC2 (S011477) | T | 8p21.2 | NEFL (S011416) | T | 11q13.3 | CCND1 (S011422) | T |
| 1p32.3 | OSBPL9 (S011024) | K | 3p26.2 | CRBN (S011401) | T | 5q35.3 | COL23A1 (S013387) | K | 8p21.2 | CDCA2 (S011064) | K | 11q22 | MTMR2 (S011083) | K |
| 1p32.2 | PPAP2B (S011023) | K | 3p26.1 | SUMF1 (S013175) | K | 5q35.3 | MAPK9 (S012993) | K | 8p12 | RBPMS (S011063) | K | 11q22.2 | BIRC3 (S011499) | T |
| 1p32.2 | DAB1 (S011454) | T | 3p24.2 | NR1D2 (S011035) | K | 6p25.3 | IRF4 (S013429) | T | 8p12 | GSR (S011062) | K | 11q22.2 | BIRC2 (S011424) | T |
| 1p32.2 | DAB1 (S011453) | T | 3p24.1 | NEK10 (S011034) | K | 6p25.3 | IRF4 (S011412) | T | 8p11.23 | ZNF703 (S011488) | T | 11q22.3 | ATM (S010241) | T |
| 1p31.3 | LEPR (S011451) | T | 3p12.3 | CNTN3 (S011033) | K | 6p25.2 | SERPINB6 (S013346) | K/R | 8q12.2 | CHD7 (S011490) | K/R | 11q22.3 | ATM (S011500) | T |
| 1p31.3 | RPE65 (S011452) | T | 3p11.1 | HTR1F (S011032) | K | 6p25.2 | ECI2 (S013200) | K/R | 8q12.2 | CHD7 (S011066) | K/R | 11q22.3 | ATM (S010284) | T |
| 1p21.3 | DPYD (S011450) | T | 3q12.1 | CPOX (S011036) | K | 6p22.3 | JARID2 (S011479) | T | 8q21.3 | RMDN1 (S011068) | K/R | 11q22.3 | ATM (S010293) | T |
| 1p21.1 | COL11A1 (S011449) | T | 3q23 | CLSTN2 (S011037) | K | 6p22.3 | JARID2 (S011480) | T | 8q21.3 | CPNE3 (S011067) | K/R | 11q25 | NTM (S013142) | K |
| 1p12 | FAM46C (S011448) | T | 3q23 | ATR (S011469) | T | 6p22.3 | KIAA0319 (S011411) | T | 8q22.3 | RRM2B (S011491) | T | 11q25 | JAM3 (S011426) | T |
| 1p12 | FAM46C (S011386) | T | 3q23 | ATR (S013431) | T | 6p22.1 | ZFP57 (S011051) | K/R | 8q24.21 | MYC (S011492) | T | 11q25 | NCAPD3 (S011425) | T |
| 1p12 | SPAG17 (S011022) | K | 3q23 | ATR (S011468) | T | 6p21.33 | TNF (S011410) | K/R | 8q24.21 | MYC (S011493) | T | 11q25 | NCAPD3 (S013089) | K |
| 1q21.1 | PDZK1 (S011388) | T | 3q24 | SLC9A9 (S011038) | K | 6p12.3 | PKHD1 (S011478) | K/R | 8q24.21 | MYC (S010805) | T | 12p13.33 | WNK1 (S013063) | K |
| 1q21.2 | BCL9 (S011389) | T | 3q29 | ACAP2 (S013332) | K | 6p11.2 | PRIM2 (S011050) | K/R | 8q24.3 | SLC39A4 (S012965) | K/R | 12p13.33 | CACNA2D4 (S013116) | K |
| 1q21.2 | ANP32E (S011391) | T | 3q29 | KIAA0226 (S013000) | K | 6q12 | EYS (S011481) | T | 9p24.3 | DOCK8 (S013022) | K | 12p13.33 | TSPAN9 (S011505) | K |
| 1q21.2 | ANP32E (S011390) | T | 4p16.3 | FGFR3 (S011404) | T | 6q13 | COL19A1 (S011052) | K | 9p24.1 | JAK2 (S011494) | T | 12p13.31 | LTBR (S011432) | T |
| 1q21.3 | RPRD2 (S011025) | K | 4p16.3 | FGFR3 (S011405) | T | 6q13 | RIMS1 (S011053) | K | 9p24.1 | GLDC (S013009) | K | 12p13.31 | LTBR (S011431) | T |
| 1q21.3 | MCL1 (S011394) | T | 4p16.3 | FGFR3 (S011403) | T | 6q22.33 | LAMA2 (S011054) | K | 9p22.3 | FREM1 (S011072) | K | 12p13.31 | CD27 (S011503) | T |
| 1q21.3 | MCL1 (S011396) | T | 4p16.3 | LETM1 (S013364) | K/R | 6q23.3 | TNFAIP3 (S011482) | T | 9p22.2 | BNC2 (S011071) | K | 12p13.31 | VAMP1 (S011428) | T |
| 1q21.3 | NUP210L (S011026) | K | 4p16.3 | WHSC1 (S011402) | T | 6q25.3 | TFB1M (S011414) | T | 9p13.2 | FBXO10 (S011069) | K | 12p13.31 | NCAPD2 (S011429) | T |
| 1q21.3 | ADAR (S011392) | T | 4p16.3 | WHSC1 (S011406) | T | 6q25.3 | WTAP (S011483) | T | 9p13.2 | DCAF10 (S011070) | K | 12p13.31 | NCAPD2 (S011427) | T |
| 1q21.3 | ADAR (S011393) | T | 4p16.3 | ADD1 (S013059) | K/R | 6q25.3 | IGF2R (S011413) | T | 9q21.12 | TRPM3 (S011073) | K | 12p13.31 | CHD4 (S011430) | T |
| 1q21.3 | CKS1B (S011459) | T | 4p15.32 | LDB2 (S000062) | K/R | 6q26 | PARK2 (S011484) | T | 9q31.1 | ALDOB (S011074) | K | 12p13.31 | CHD4 (S011504) | T |
| 1q21.3 | CKS1B (S011460) | T | 4p15.31 | KCNIP4 (S011039) | K/R | 6q26 | PARK2 (S011415) | T | 9q34.3 | COL5A1 (S011495) | T | 12p13.2 | ETV6 (S011502) | T |
| 1q21.3 | CKS1B (S011395) | T | 4p13 | ATP8A1 (S011470) | K/R | 6q27 | SMOC2 (S013056) | K | 9q34.3 | TRAF2 (S011419) | T | 12p13.1 | CDKN1B (S011501) | T |
| 1q23.3 | SLAMF7 (S011399) | T | 4q13.1 | TECRL (S011040) | K/R | 6q27 | ERMARD (S013199) | K | 9q34.3 | TRAF2 (S011420) | T | 12p12.3 | AEBP2 (S011086) | K |
| 1q23.3 | SLAMF7 (S011398) | T | 4q13.2 | UGT2A1 (S011041) | K/R | 7p22.1 | RADIL (S013184) | K | 9q34.3 | GRIN1 (S013195) | K | 12p11.22 | FAR2 (S011084) | K/R |
| 1q23.3 | NUF2 (S011397) | T | 4q25 | CFI (S011471) | K/R | 7p15.3 | RAPGEF5 (S011057) | K | 9q34.3 | EHMT1 (S013020) | K | 12p11.22 | TMTC1 (S011085) | K/R |
| 1q23.3 | RP11 (S011463) | T | 4q31.22 | ZNF827 (S011042) | K/R | 7p15.3 | STK31 (S011058) | K | 10p15.3 | DIP2C (S013412) | K/R | 12q12 | KIF21A (S010040) | K/R |
| 1q23.3 | RP11 (S011466) | T | 4q35.2 | CYP4V2 (S013202) | K/R | 7p12.3 | ADCY1 (S011055) | K | 10p15.2 | PFKP (S013187) | K/R | 12q12 | NELL2 (S014297) | K/R |
| 1q23.3 | RP11 (S011465) | T | 4q35.2 | TRIML1 (S012955) | K/R | 7p12.3 | ABCA13 (S011056) | K | 10p14 | UPF2 (S011496) | K/R | 12q23.1 | NEDD1 (S011088) | K/R |
| 1q23.3 | RP11 (S011464) | T | 5p15.33 | IRX4 (S013194) | K | 7p12.2 | IKZF1 (S013432) | T | 10p13 | NMT2 (S011076) | K/R | 12q23.1 | SLC17A8 (S011087) | K/R |
| 1q23.3 | RP11 (S011462) | T | 5p15.31 | NSUN2 (S013190) | K | 7p12.2 | IKZF1 (S011485) | T | 10p13 | ITGA8 (S011077) | K/R | 12q24.22 | NOS1 (S011507) | K/R |
| 1q23.3 | PBX1 (S011461) | T | 5p15.2 | DNAH5 (S011046) | K | 7p12.2 | IKZF1 (S011486) | T | 10p11.1 | ZNF25 (S011075) | K/R | 12q24.33 | GALNT9 (S013154) | K/R |
| 1q31.3 | KCNT2 (S011027) | K | 5p13.3 | NPR3 (S011045) | K | 7p11.2 | LANCL2 (S014303) | K | 10q11.21 | MARCH8 (S011078) | K/R | 12q24.33 | PGAM5 (S013304) | K/R |
| 1q44 | ADSS (S013049) | K | 5p13.2 | TTC23L (S011044) | K | 7q11.22 | WBSCR17 (S011059) | K | 10q11.22 | ARHGAP22 (S011079) | K/R | 13q12.3 | KATNAL1 (S011089) | K |
| 1q44 | DESI2 (S013025) | K | 5q11.2 | IL31RA (S011048) | K | 7q31.1 | PNPLA8 (S011060) | K | 10q22.2 | KAT6B (S011080) | K/R | 13q14.11 | ENOX1 (S011537) | T |
| 2p25.3 | TMEM18 (S012958) | K/R | 5q11.2 | MIER3 (S011047) | K | 7q31.1 | IFRD1 (S011061) | K | 10q25.1 | ADD3 (S011421) | K/R | 13q14.2 | RB1 (S011434) | T |
| 2p25.3 | COLEC11 (S013217) | K/R | 5q31.2 | MYOT (S011049) | T | 7q34 | BRAF (S011487) | T | 10q26.3 | INPP5A (S013003) | K/R | 13q14.2 | RB1 (S011509) | T |
| 2p22.3 | SPAST (S011029) | K/R | 5q31.2 | CTNNA1 (S011472) | T | 7q36.3 | RBM33 (S013182) | K | 10q26.3 | KNDC1 (S012996) | K/R | 13q14.2 | RCBTB2 (S011510) | T |
| 2p16.1 | PEX13 (S011400) | K/R | 5q31.3 | PCDHA1 (S011473) | T | 7q36.3 | WDR60 (S012950) | K | 11p15.5 | RIC8A (S013302) | K | 13q14.2 | DLEU2 (S011508) | T |

| hg19 location | | gene  (probe number) | type | | hg19 location | | gene  (probe number) | | type | hg19 location | gene  (probe number) | | type | |
| --- | --- | --- | --- | --- | --- | --- | --- | --- | --- | --- | --- | --- | --- | --- |
| 13q14.2 | KCNRG (S011538) | | | T | | 16q23.2 | | MAF (S011443) | T | 19q13.11 | | SLC7A9 (S011116) | | K |
| 13q14.2 | MIR15A (S011539) | | | T | | 16q24.3 | | ANKRD11 (S013330) | K | 19q13.42 | | DNAAF3 (S013156) | | K |
| 13q14.2 | DLEU1 (S011435) | | | T | | 16q24.3 | | GAS8 (S013152) | K | 19q13.43 | | SLC27A5 (S013344) | | K |
| 13q14.2 | DLEU1 (S011541) | | | T | | 17p13.3 | | VPS53 (S013064) | K/R | 20p13 | | RSPO4 (S013131) | | K/R |
| 13q14.3 | DLEU7 (S011543) | | | T | | 17p13.3 | | NXN (S013141) | K/R | 20p13 | | TGM6 (S013070) | | K/R |
| 13q14.3 | RNASEH2B (S011511) | | | T | | 17p13.1 | | TP53 (S010576) | T | 20p12.3 | | TRMT6 (S011121) | | K/R |
| 13q14.3 | ATP7B (S011540) | | | T | | 17p13.1 | | TP53 (S010577) | T | 20p12.2 | | PLCB4 (S011120) | | K/R |
| 13q14.3 | VPS36 (S014299) | | | T | | 17p13.1 | | TP53 (S010578) | T | 20p11.23 | | RIN2 (S011119) | | K/R |
| 13q14.3 | PCDH8 (S011542) | | | T | | 17p13.1 | | TP53 (S010580) | T | 20q11.22 | | ACSS2 (S011123) | | K/R |
| 13q21.33 | KLHL1 (S012572) | | | K | | 17p13.1 | | TP53 (S010581) | T | 20q11.22 | | EDEM2 (S011122) | | K/R |
| 13q22.1 | DIS3 (S011436) | | | T | | 17p13.1 | | TP53 (S010582) | T | 20q11.23 | | SAMHD1 (S011527) | | K/R |
| 13q22.1 | DIS3 (S011437) | | | T | | 17p13.1 | | TP53 (S010583) | T | 20q12 | | MAFB (S011447) | | T |
| 13q34 | ARHGEF7 (S013121) | | | K | | 17p13.1 | | TP53 (S010584) | T | 20q13.12 | | SLC13A3 (S011124) | | K/R |
| 13q34 | GRK1 (S013151) | | | K | | 17p13.1 | | TP53 (S010585) | T | 20q13.13 | | STAU1 (S011125) | | K/R |
| 14q11.2 | CHD8 (S011090) | | | K/R | | 17p13.1 | | TP53 (S010586) | T | 20q13.33 | | OSBPL2 (S013088) | | K/R |
| 14q22.1 | DDHD1 (S011091) | | | K/R | | 17p13.1 | | TP53 (S010587) | T | 20q13.33 | | UCKL1 (S013125) | | K/R |
| 14q22.2 | SAMD4A (S011092) | | | K/R | | 17p13.1 | | TP53 (S010588) | T | 21q11.2 | | RBM11 (S013183) | | K |
| 14q24.3 | NPC2 (S011512) | | | K/R | | 17p13.1 | | TP53 (S010589) | T | 21q11.2 | | HSPA13 (S011126) | | K |
| 14q32.31 | DYNC1H1 (S013378) | | | K/R | | 17p13.1 | | TP53 (S010590) | T | 21q22.11 | | ITSN1 (S011127) | | K |
| 14q32.32 | TRAF3 (S011513) | | | T | | 17p13.1 | | PIK3R6 (S011104) | K/R | 21q22.2 | | PSMG1 (S011528) | | K |
| 14q32.32 | TRAF3 (S013430) | | | T | | 17p13.1 | | USP43 (S011103) | K/R | 21q22.3 | | PDE9A (S013140) | | K |
| 14q32.33 | APOPT1 (S013391) | | | K/R | | 17p11.2 | | RAI1 (S011102) | K/R | 21q22.3 | | PWP2 (S011128) | | K |
| 14q32.33 | CEP170B (S011440) | | | T | | 17p11.2 | | MIR33B (S011522) | T | 21q22.3 | | TSPEAR (S013172) | | K |
| 14q32.33 | MTA1 (S011439) | | | T | | 17q11.2 | | PSMD11 (S011105) | K/R | 22q11.1 | | GAB4 (S014304) | | T |
| 14q32.33 | MTA1 (S011514) | | | T | | 17q12 | | IKZF3 (S011444) | T | 22q11.21 | | CECR2 (S014302) | | T |
| 14q32.33 | IGHD (S011441) | | | T | | 17q12 | | IKZF3 (S011523) | T | 22q11.21 | | HIRA (S011529) | | T |
| 15q12 | GABRB3 (S011515) | | | K | | 17q21.31 | | MAP3K14 (S011446) | T | 22q11.23 | | SMARCB1 (S011530) | | T |
| 15q12 | GABRB3 (S011093) | | | K | | 17q21.31 | | MAP3K14 (S011445) | T | 22q11.23 | | SMARCB1 (S011531) | | T |
| 15q22.2 | VPS13C (S011094) | | | K | | 17q23.2 | | MED13 (S011106) | K/R | 22q12.2 | | NF2 (S011532) | | T |
| 15q22.31 | USP3 (S011095) | | | K | | 17q25.3 | | CCDC57 (S013115) | K/R | 22q12.2 | | ZMAT5 (S014300) | | K |
| 15q26.3 | IGF1R (S011516) | | | T | | 17q25.3 | | CSNK1D (S013157) | K/R | 22q12.2 | | SFI1 (S014298) | | K |
| 15q26.3 | CHSY1 (S013163) | | | K | | 18p11.31 | | LPIN2 (S011109) | K/R | 22q12.3 | | LARGE (S011533) | | T |
| 15q26.3 | TM2D3 (S013299) | | | K | | 18p11.31 | | TGIF1 (S011525) | K/R | 22q13.2 | | EP300 (S011534) | | T |
| 16p13.3 | DECR2 (S013415) | | | K/R | | 18p11.21 | | GNAL (S011108) | K/R | 22q13.31 | | TRMU (S013069) | | K |
| 16p13.3 | IFT140 (S013104) | | | K/R | | 18p11.21 | | SPIRE1 (S011107) | K/R | 22q13.33 | | BRD1 (S013118) | | K |
| 16p13.13 | TXNDC11 (S011098) | | | K/R | | 18p11.21 | | RNMT (S011524) | K/R |  | |  | |  |
| 16p13.12 | CPPED1 (S011097) | | | K/R | | 18q11.2 | | NPC1 (S011526) | K/R |  | |  | |  |
| 16p11.2 | HIRIP3 (S011096) | | | K/R | | 18q11.2 | | NPC1 (S011110) | K/R |  | |  | |  |
| 16q11.2 | GPT2 (S010065) | | | K | | 18q21.1 | | LOXHD1 (S011111) | K/R |  | |  | |  |
| 16q12.1 | LONP2 (S011099) | | | K | | 18q21.1 | | LIPG (S011112) | K/R |  | |  | |  |
| 16q12.1 | CYLD (S011442) | | | T | | 18q23 | | CTDP1 (S013109) | K/R |  | |  | |  |
| 16q12.1 | CYLD (S011517) | | | T | | 18q23 | | TXNL4A (S013126) | K/R |  | |  | |  |
| 16q13 | SLC12A3 (S011518) | | | T | | 19p13.3 | | PPAP2C (S013083) | K |  | |  | |  |
| 16q22.1 | SLC12A4 (S011100) | | | K | | 19p13.3 | | CDC34 (S013220) | K |  | |  | |  |
| 16q22.1 | DUS2 (S011101) | | | K | | 19p13.2 | | GCDH (S011115) | K |  | |  | |  |
| 16q23.1 | WWOX (S011520) | | | T | | 19p13.2 | | STX10 (S011114) | K |  | |  | |  |
| 16q23.1 | WWOX (S011521) | | | T | | 19p13.11 | | GMIP (S011113) | K |  | |  | |  |

**Supplementary Table 4: Correlation of scaled Schoenfeld residuals with time for each covariate of the multivariate cox regression model.**

|  | Chisq | *p-value* |
| --- | --- | --- |
| t(4;14) | 0.003 | 0.96 |
| t(14;16) | 8.13 | 0.0043 |
| Gain(1q) | 0.31 | 0.58 |
| Del(1p)/*CDKN2C* | 0.28 | 0.60 |
| Gain(*MYC*) | 1.33 | 0.25 |
| Del(17p) | 0.50 | 0.48 |
| TNE | 0.25 | 0.62 |
| Global | 12.78 | 0.07 |
