# Supplementary material for: Copy number evolution and its relationship with patient outcome—an analysis of 178 matched presentation-relapse tumor pairs from the Myeloma XI trial
Source: Leukemia. 2020 Dec 1;35(7):2043–53. doi: 10.1038/s41375-020-01096-y (PMC8257500; doi:10.1038/s41375-020-01096-y)

**SUPPLEMENTARY FIGURES**

**Supplementary Figure 1. Treatment pathways of 178 patients included in the analysis.** TE = Transplant eligible; TNE= Transplant non eligible; CRD = Cyclophosphamide, Lenalidomide, Dexamethasone; CTD = Cyclophosphamide, Thalidomide, Dexamethasone; KCRD= Carfilzomib, Cyclophosphamide, Lenalidomide, Dexamethasone; a=attenuated; CVD= Cyclophosphamide, Bortezomib, Dexamethasone; HDM =High dose Melphalan; Len = Lenalidomide; Len V = Lenalidomide, Vorinostat; Ob =observation; NR = Not Randomised. For analysis purposes, patients randomized to lenalidomide or lenalidomide and vorinostat were grouped together as ‘lenalidomide maintenance’.


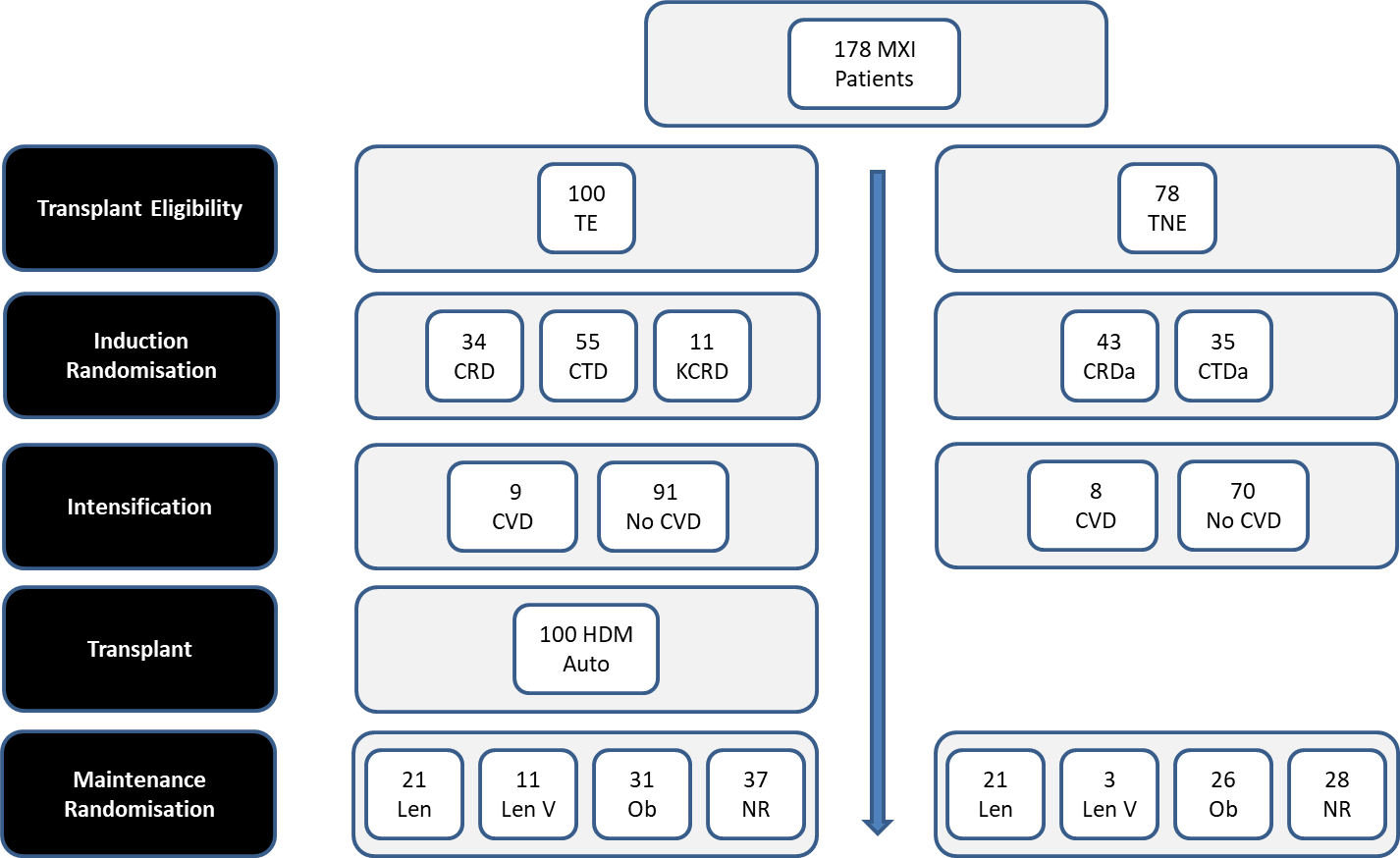


**Supplementary Figure 2. Frequency of presentation CNAs lost at relapse.** Frequency of chromosome arm CNAs reverting to diploid at relapse from diagnostic aberration: gain (red), amplification (yellow), heterozygous deletion (blue) or homozygous deletion (green).


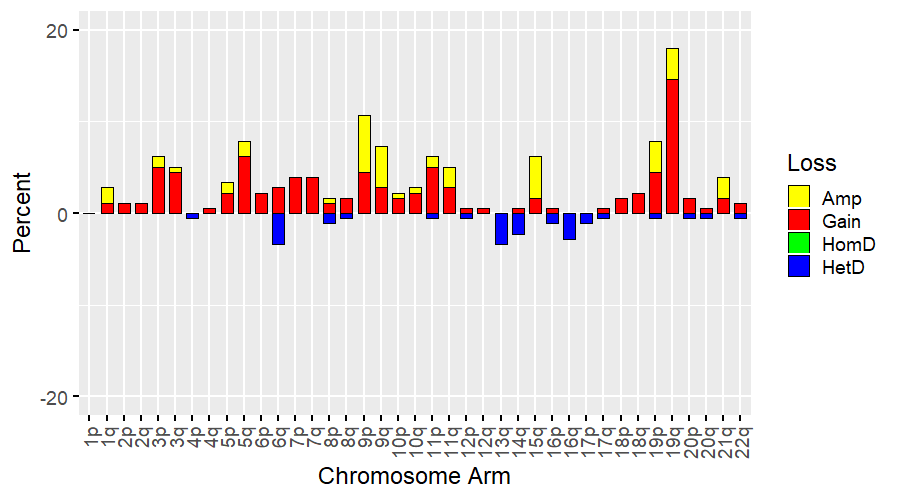


**Supplementary Figure 3. Focal CNA evolution in IMiD response genes.** Number of individual tumors with emergence of focal CNAs in IMiD response genes (upper part) or their reversal from abnormal to diploid (lower part).


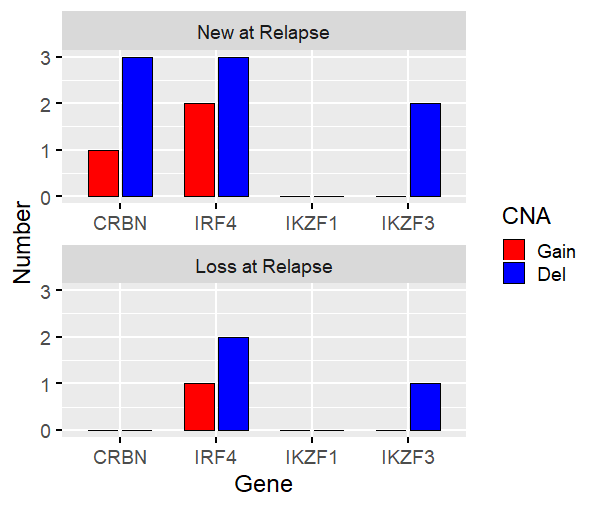


**Supplementary Figure 4. Sub-clonal to clonal emergence of CNAs (A, B) gain(1q) and (C, D) del(17p).**  Clonal gain(1q) or del(17p) was considered in relapse tumors when the majority (>50%) of relevant dMLPA probes had normalised values of >1.2 or ≤0.75 respectively. The potential presence of sub-clonal aberrations in presentation tumors was considered when the majority of relevant dMLPA probes were >1.05 ≤1.20 for gain(1q) and >0.75 ≤0.95 for del(17p). Left hand side: scatter plots of normalised values of relevant dMLPA probes at presentation (x axis) and relapse (y axis). Right hand side: density plots of normalised values of relevant dMLPA probes at presentation. Pink shading indicates tumors with probes values sitting within sub-clonal range at presentation.


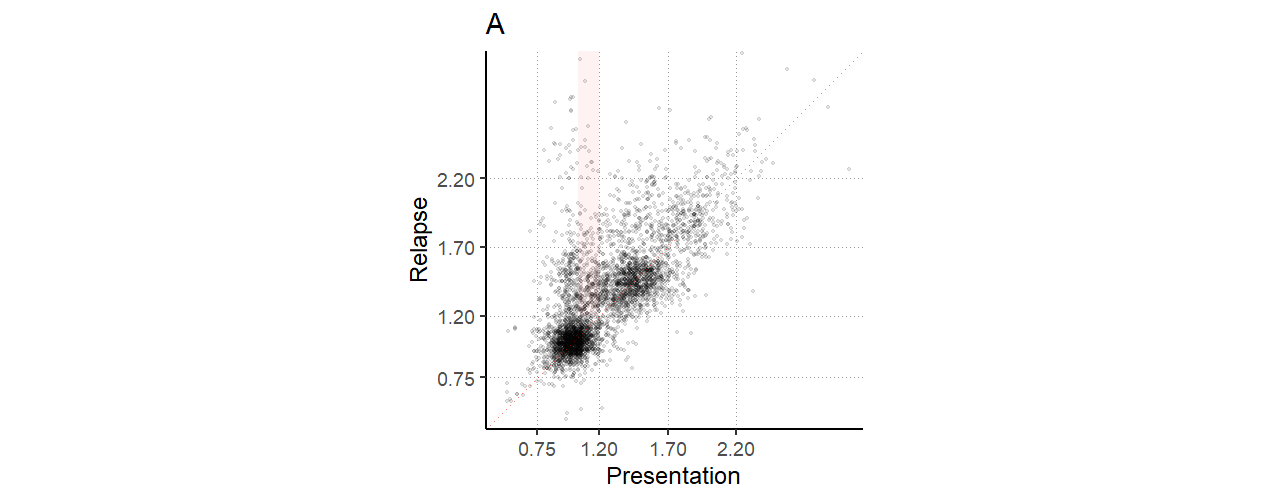

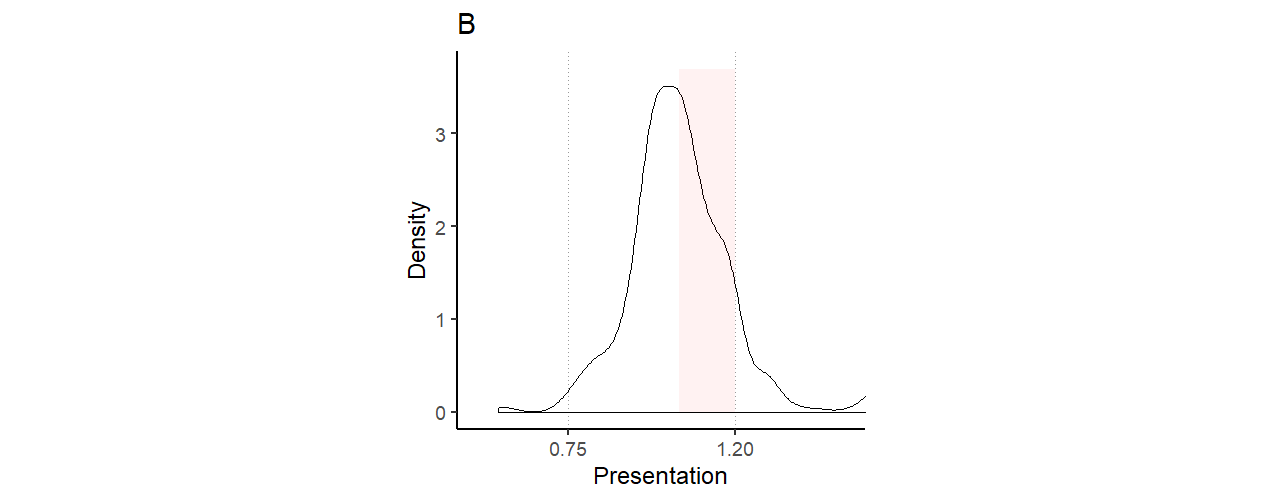


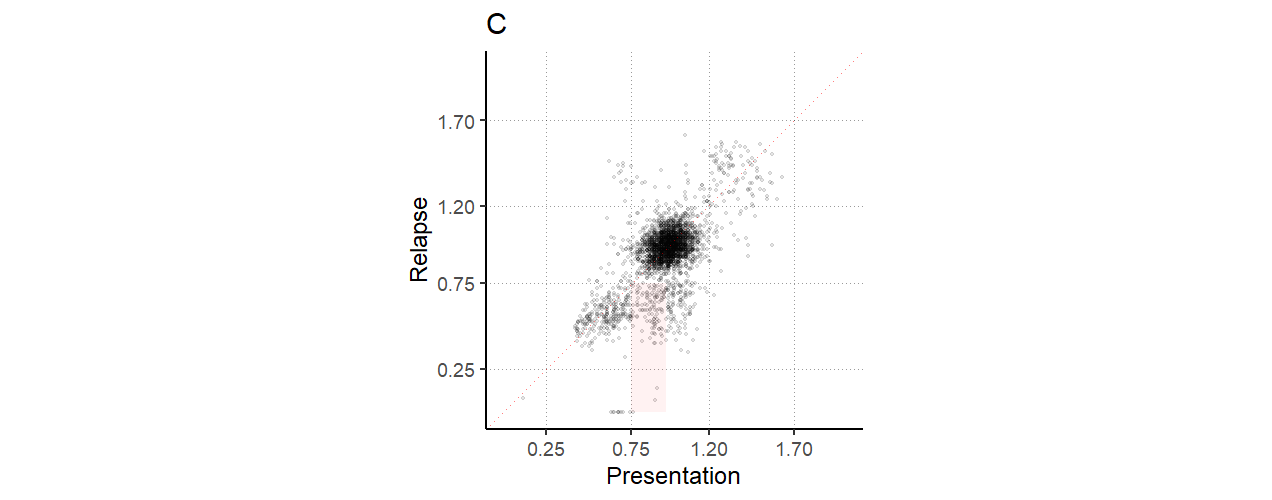

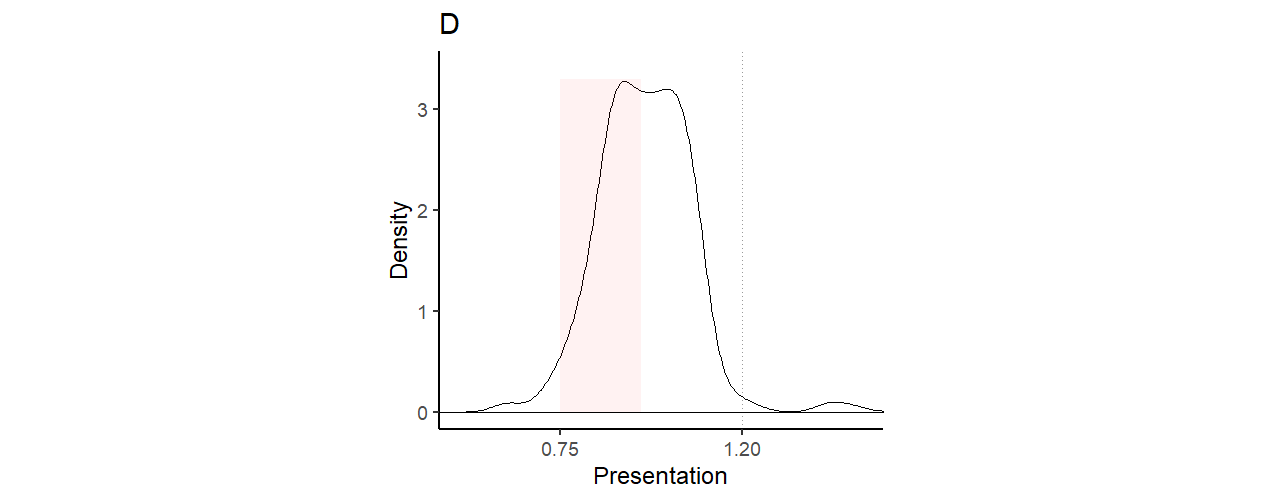


**Supplementary Figure 5. Comparison of frequency of CNAs at presentation and relapse per major molecular subgroups. (A)** gain(1q)/amp(1q) **(B)** del(13q) **(C)** del(17p)


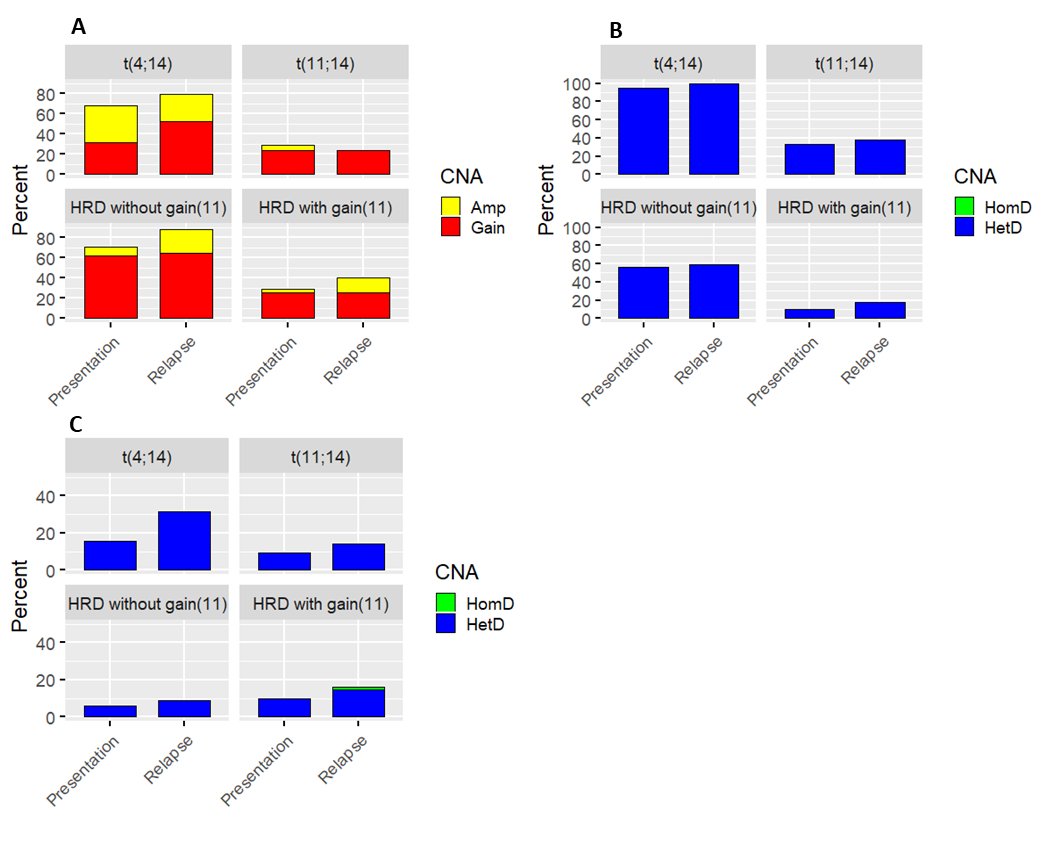


**Supplementary Figure 6. Whole arm CNA frequency within t(14;16) and t(14;20) subgroups at baseline and relapse.** t(14;16) subgroup (2 patients), baseline (A), relapse (B). t(14;20) subgroup (1 patient), baseline (C), relapse (D)


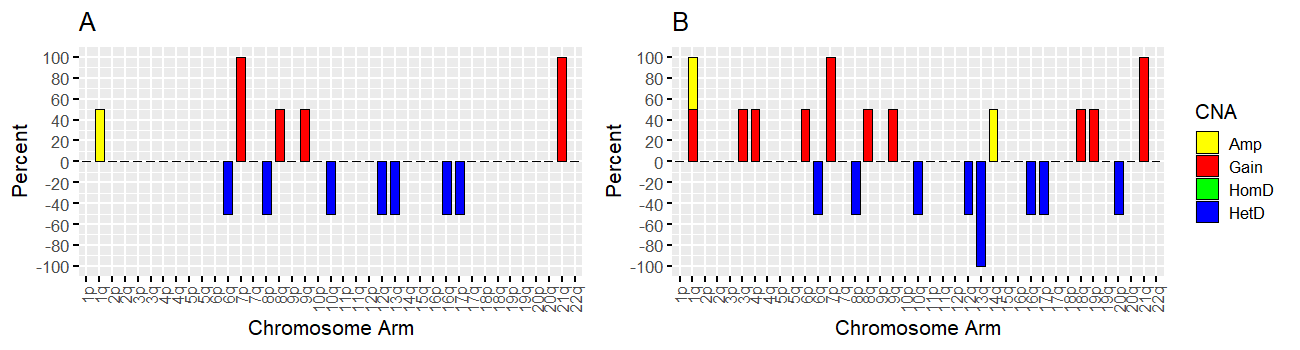

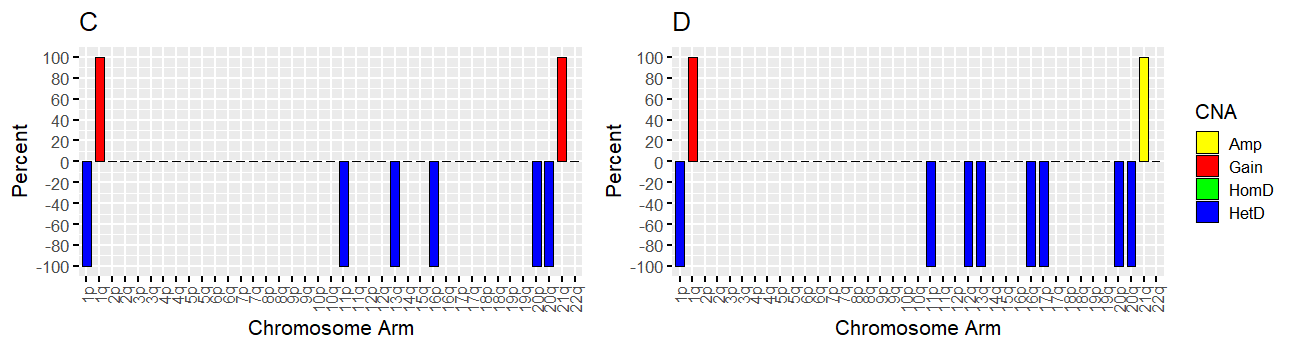
.

**Supplementary Figure 7. Molecular tumor characterization based on Cyclin D expression patterns.** Heatmap of *CCND1* and *CCND2* expression patterns (green color coded bars, middle heatmap) in context of presentation CNAs (right heatmap) and IG translocations (left). For 158 tumors with available qRT-PCR results, unsupervised clustering was applied to *CCND1 and CCND2* expression values and three clusters with predominant *CCND1* (D1; n=78), *CCND2* (D2; n=55) or co-occurring expression (D1+D2; n=25) segregated, which were annotated with molecular profiles. Color-coding for individual elements of the diagram: (left) black = present; grey = absent; (middle) relative qRT-PCR expression values with white = low expression to dark green = high expression; (right) normalized digitalMLPA copy number ratios with 1.0 equivalent to normal/diploid (grey), red = copy number gain, blue = copy number loss. Legend bottom: black and white bands representing chromosomal mapping of digitalMLPA probes chr1-22 from left to right in ascending order of genomic position.


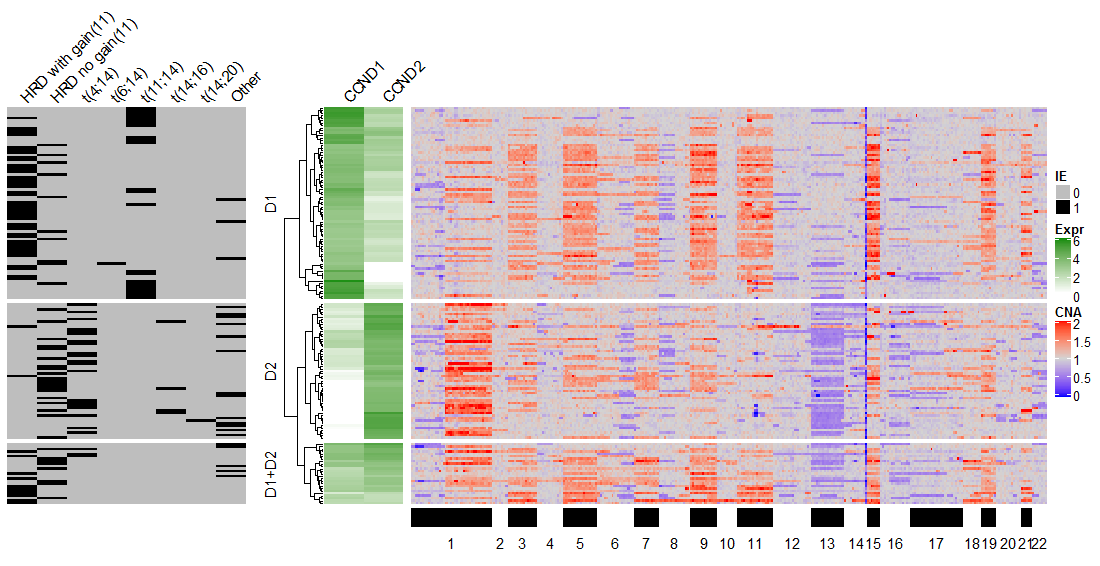


**Supplementary Figure 8.** **Comparison of frequency of CNA between presentation and relapse per *CCND* expression group.** **(A)** for gain(1q)/amp(1q), **(B)** for heterozygous/homozygous del(17p).


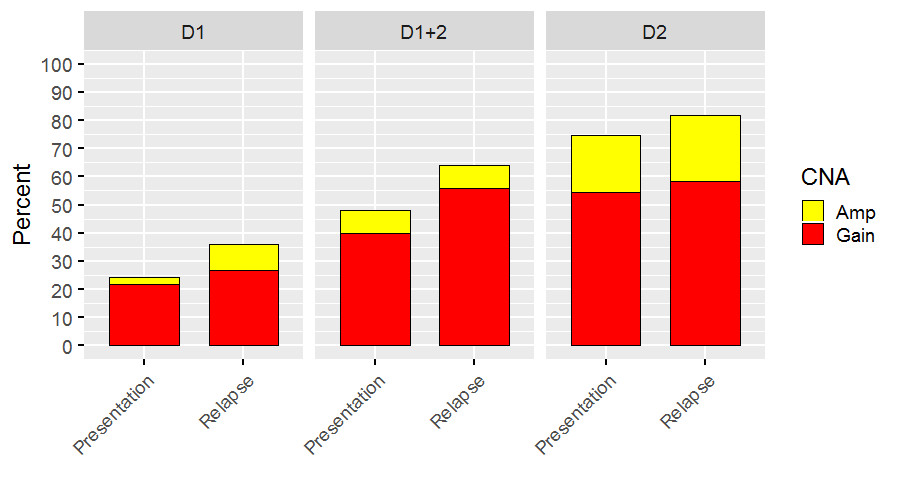


**B)**
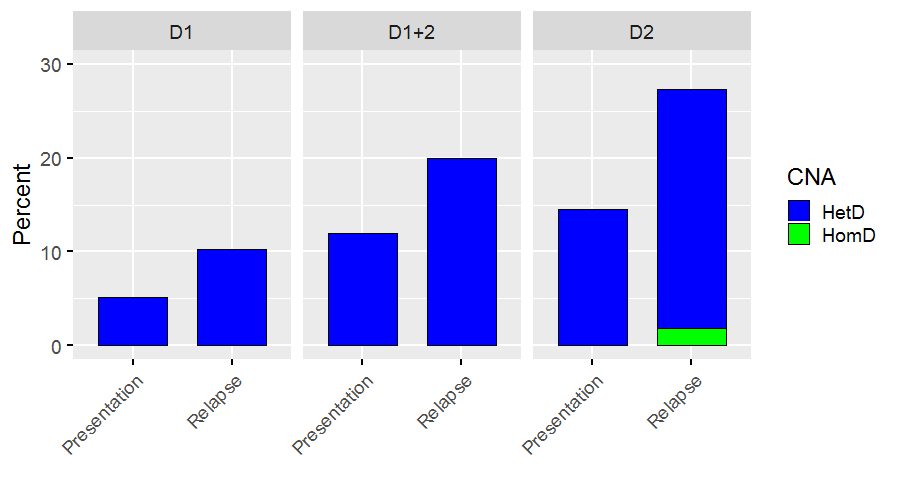


**Supplementary Figure 9. Frequency of new chromosome CNA evolution at relapse**. (A) HRD without gain(11), (B) HRD with gain(11).

**A)**


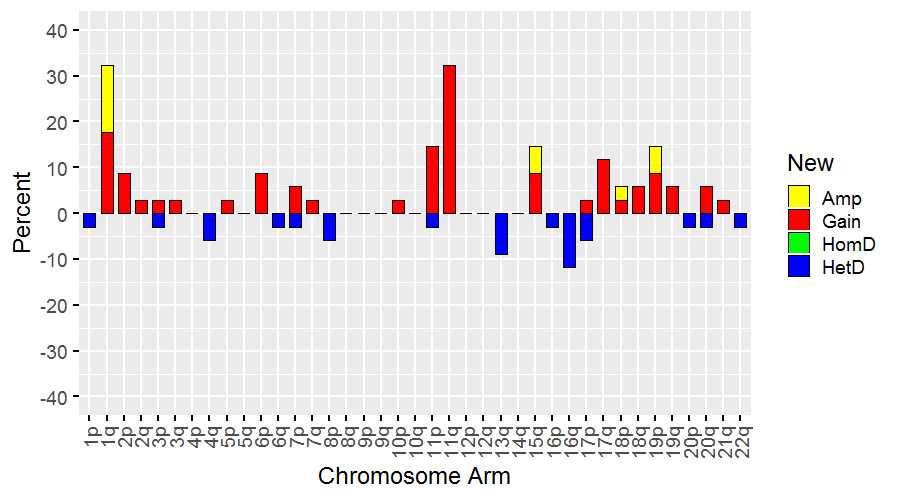


**B)**


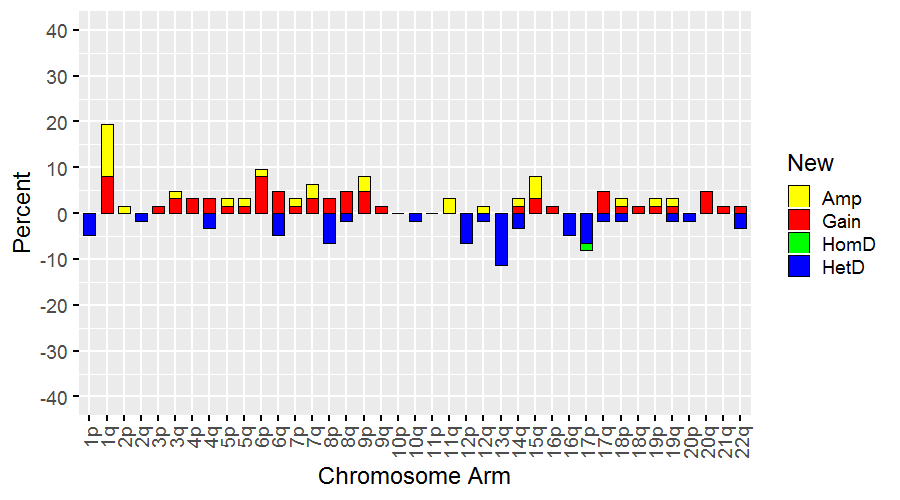


**Supplementary Figure 10. Graphical representation of proportional hazards assumption for each time dependent covariate within the multivariate regression model.** Plots demonstrates each covariate’s log hazard ratio (HR) over time (depicted by the solid black line), each plot shows a relatively flat horizontal trajectory indicating stable HR regardless of time of covariate acquisition. Dashed black lines represent 95% confidence intervals (CI) of HR over time and dashed green line average hazard ratio (HR) over time. The circles represent Schoenfeld’s residuals, the proportional hazards assumption is upheld for each covariate, demonstrated by absence of significant correlation between residuals and time. Widening CIs to the right of plots reflect increasing uncertainty due to lower number of cases with respective follow-up.


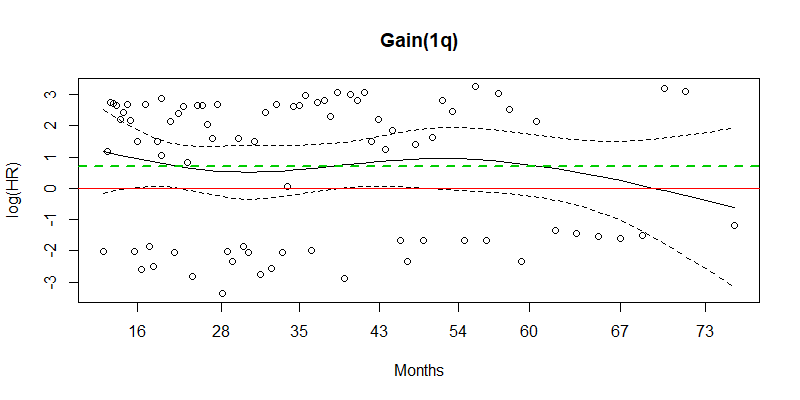

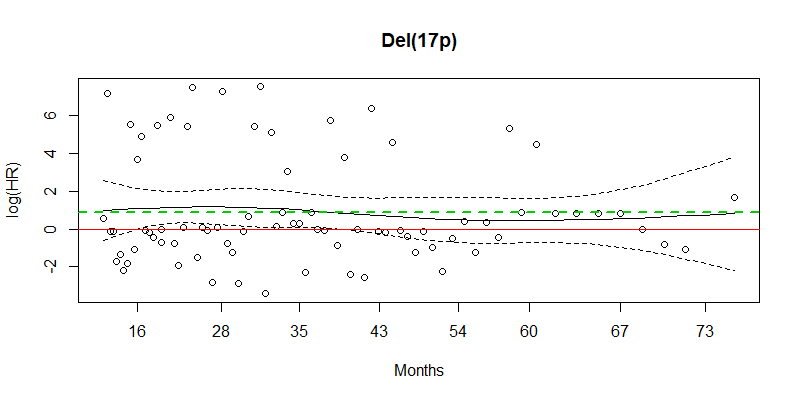

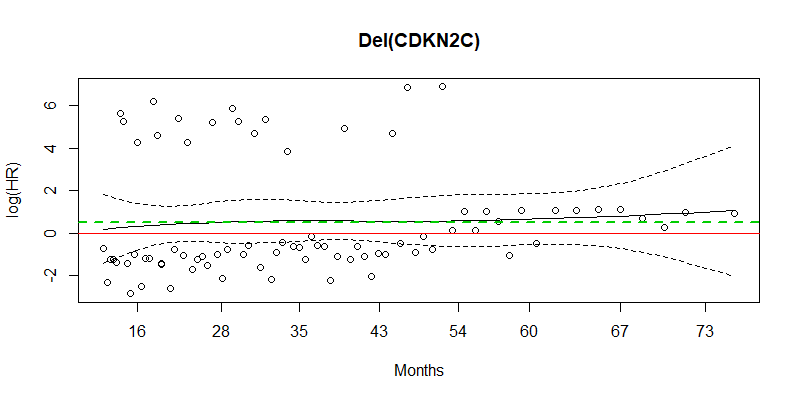

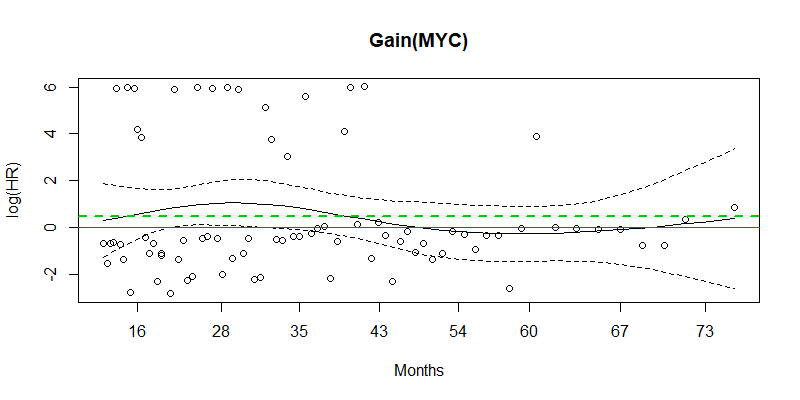

Supplement: Supplementary file 3 — Supplementary Figures [file 41375_2020_1096_MOESM3_ESM.docx]
